# Mononeuritis Multiplex Presenting with Retroperitoneal Inflammation: A Case-Based Review in the Light of Diagnostic and Therapeutic Challenges

**DOI:** 10.31138/mjr.280725.ecz

**Published:** 2026-03-01

**Authors:** Michail-Angelos Mourtzos, Natalia-Maria Keklikoglou, Dimitrios I. Patoulias

**Affiliations:** 1 School of Medicine, Faculty of Health Sciences, Aristotle University of Thessaloniki, Thessaloniki, Greece;; 2 Department of Physiotherapy, Faculty of Health Sciences, International Hellenic University, Alexander Campus, Thessaloniki, Greece;; 3 Second Propaedeutic Department of Internal Medicine, Faculty of Medicine, School of Health Sciences, Aristotle University of Thessaloniki, Thessaloniki, Greece

**Keywords:** mononeuritis multiplex, retroperitoneal inflammation, vasculitic neuropathy, immune-mediated neuropathy

## Abstract

**Background:**

Mononeuritis multiplex (MNM), is an uncommon, peripheral neuropathy, presenting with asymmetric involvement of multiple individual nerves. It is frequently associated with vasculitic disorders and other immune-related conditions. Early manifestations can often be non-specific, which might complicate diagnosis.

**Case presentation:**

A 56-year-old woman with no significant medical history developed sudden abdominal and right gluteal pain followed by progressive and asymmetric sensorimotor deficits. Initial imaging demonstrated fat stranding and infiltration in the retroperitoneum, lateral pelvic walls, and lumbosacral plexus, suggestive of an inflammatory process. Laboratory testing demonstrated marked leucocytosis, eosinophilia, elevated erythrocyte sedimentation rate (ESR) and C-reactive protein (CRP), sterile pyuria, hypoalbuminemia and elevated alpha- and beta-globulins. Markers of infectious and autoimmune diseases, as well as neoplastic markers were unrevealing. Nerve biopsy was inconclusive. Therefore, a diagnosis of immune-mediated vasculitic neuropathy was favoured but not confirmed. She was treated with high-dose oral corticosteroids tapered over 12 months, with additional antibiotics to reduce risk of infection. At follow-up, she remained ambulatory and independent, with mild residual deficits and intermittent inflammatory marker elevation.

**Conclusions:**

This case illustrates how MNM presenting with gradual evolution, mixed infectious and autoimmune features, and in the context of retroperitoneal inflammation, can complicate the diagnostic process. In absence of biopsy confirmation, management required balancing empirical immunosuppression with infection risk. Comparison with prior reports underlines the importance of complete autoimmune workup, biopsy and imaging techniques such as positron emission tomography scan in minimising diagnostic uncertainty.

## INTRODUCTION

Mononeuritis multiplex (MNM) is a rare type of peripheral neuropathy, characterised by acute or subacute onset of painful asymmetric sensorimotor deficits involving multiple non-contiguous peripheral nerves. In later stages, the disease may progress and eventually resemble generalised polyneuropathy, thus obscuring the initial diagnostic pattern.^1^ Among the most common causes are vasculitis, and autoimmune connective tissue diseases (CTD), ^2^ while infectious agents (e.g., hepatitis B/C, human immunodeficiency virus, leprosy), paraneoplastic syndromes, hematologic malignancies, drug toxicity, and diabetes mellitus have also been associated with MNM.^1^ Neurophysiologic studies (NCS) and electromyography (EMG) are very important parts of the investigation, as they can reveal MNM’s characteristic pattern of nerve damage (axonal neuropathy).^1,2^

However, beyond well-defined systemic disorders, MNM can present with vague presentations or overlapping features and therefore pose a diagnostic challenge. Such atypical forms have been described in vasculitis, autoimmune connective tissue diseases, malignancy and even in IgG4-related disease.^3–7^ In sight of diagnostic uncertainty, histologic confirmation can provide valuable insight regarding the underlying aetiology, even though it is often missed during the diagnostic workup.^8^

In that context, we present an ambiguous case of a 56-year-old woman who presented with MNM, preceded by severe abdominal and gluteal pain and retroperitoneal inflammation. The staggered symptom evolution, inconclusive findings, and lack of definitive diagnosis underscore the complexities of MNM evaluation and the need for a multidisciplinary approach in atypical presentations.

## CASE DESCRIPTION

### Patient Information

A 56-year-old woman, with no relevant medical or family history presented with acute lower abdominal and right gluteal pain, followed by sudden onset asymmetric sensorimotor deficits progressively affecting multiple peripheral nerves. Early imaging revealed retroperitoneal inflammatory infiltration and laboratory work-up showed persistent leucocytosis and elevated inflammatory markers. Serologic evaluation of autoimmune, infectious and metabolic diseases was unrevealing. Nerve conduction studies (NCS) were consistent with mononeuritis multiplex which, collectively with the overall course, raised suspicion of an underlying immune-mediated vasculitic aetiology.

### Clinical Findings

Patient’s history began on 04.02.2019 with acute lower abdominal pain radiating to the lumbar region, which was attributed to retroperitoneal inflammation. She was afebrile and reported no systemic complaints. Initial treatment included empirical antibiotics, leading to partial pain improvement. The patient was discharged on 07.02.2019. A day after, she experienced a transient febrile wave of 38.5 °C and on 13.02.2019, she developed acute right gluteal pain and sudden-onset right lower extremity paresis, prompting admission to a neurosurgery department. She subsequently developed gradual, progressive weakness of the left lower extremity. Cognition, cranial nerves and cerebellar function were normal. Detailed findings from the neurological examination are presented in **Table 1**. She was transferred to the neurology department and after a total of two weeks and, in the absence of a definitive diagnosis, opted for discharge against medical advice on 26.02.2019, being afebrile, with normal cognitive function and consciousness, but no improvement regarding sensorimotor deficits. Evaluation in April showed partial resolution of neurologic impairments in both lower extremities (**Table 1).** Cognition, cranial nerves and cerebellar function remained normal. However, on 04.05.2019 the patient was admitted to an internal medicine department with new sudden-onset weakness of the left thumb, index and middle fingers (**Table 1**), alongside paresis and paraesthesias in the left median nerve distribution. Corticosteroids and adjunct antibiotic therapy were initiated, and she was discharged on 10.05.2019, without a conclusive diagnosis.

**Table 1.** Timeline of the patient’s neurologic evaluation.

**MRC scale**				
Date	02.2019	04. 2019	05.2019	05.2020
Right Lower limb				
Hip abductors	0	4	4	5
Hip adductors	0	4	4	5
Knee extensors	0	4	4	4
Knee flexors	0	4	4	4
Dorsiflexors	0	1	1	2
Plantar flexors	0	3	3	5
Instrisic foot muscles	0	1	1	2
**Left Lower limb**				
Hip abductors	4	5	5	5
Hip adductors	4	5	5	5
Knee extensors	3	4	4	5
Knee flexors	3	4	4	5
Dorsiflexors	3	4	4	5
Plantar flexors	3	4	4	5
Instrisic foot muscles	2	3	3	5
**Left Upper Arm**				
Palmar wrist flexion	5	5	3	5
Wrist extension	5	5	5	5
Finger flexion	5	5	3	4
Finger extension	5	5	5	5
Finger abduction	5	5	2	4
Finger adduction	5	5	2	4
Thumb extention	5	5	4	5

**Tendon reflexes**			
**Date**	**02.2019**	**04–05.2019**	**05.2020**
**Right lower limb**			
**Patellar**	Absent	Reduced	Lightly reduced
**Achilles**	Absent	Reduced	Lightly reduced
**Left Lower limb**			
**Patellar**	Reduced	Reduced	Normal
**Achilles**	Reduced	Reduced	Normal

### Timeline

**Table 2** outlines the full timeline of clinical course, diagnostic tests, treatments, and outcomes.

**Table 2.** Timeline of key clinical, diagnostic, and therapeutic events in the patient’s course.

**Date / Time Point**	**Clinical Events & Findings**	**Differential Diagnosis / Actions**
**Day 0–3**	Acute lower abdominal pain; leucocytosis, eosinophilia, ↑ESR, ↑CRP, retroperitoneal & presacral inflammation on CT	Suspected **infectious process** (UTI, retroperitoneal abscess); empirical broad-spectrum antibiotics initiated
**Week 1**	Onset of asymmetric motor & sensory deficits (R lower limb)	Considered infectious plexopathy due to ureteral injury
**Week 2**	Progressive multifocal deficits (R sciatic, L peroneal, L median/ulnar)	Differential expanded to **immune-mediated MNM**
**Week 2–3**	CSF analysis: normal and nerve conduction studies: multifocal, motor-predominant axonal neuropathy	Supports MNM; **GBS excluded**
**Month 2**	MRI: denervation changes in right pelvic muscles (L4–S1), resolved retroperitoneal inflammation	Plexus involvement confirmed
**Month 2–3**	Extensive serologies: negative for RF, ANA, ANCA, anti-dsDNA, anti-SSA/SSB, HIV, HCV, CMV, Toxoplasma, Wright, WIDAL, IGRA Normal: C3, C4, IgA, IgG, IgM	Autoimmune and infectious causes excluded
**Month 4**	Sural nerve biopsy inconclusive (insufficient nerve tissue sample)	Does not exclude or confirm vasculitic MNM
**Month 4–16**	Corticosteroids started & tapered over 12 months, ciprofloxacin and metformin added for prophylaxis and hyperglycaemia respectively	Clinical stabilisation with mild residual deficits
**Years 1–6**	Repeated neoplastic markers, autoimmune panels and infectious serologies: negative for CEA, CA 125, CA 15-3, CA 19-9, RF, ANA, ANCA, anti-dsDNA, anti-SSA/SSB, HIV, HCV, CMV, Toxoplasma, Wright, WIDAL, IGRA	Cancer, autoimmune and infectious markers remained negative; no disease progression; no relapses; no additional improvement regarding deficits

ESR: erythrocyte sedimentation rate; CRP: C-reactive protein; CT: computed tomography; UTI: urinary tract infection; R: right; L: left; MNM: mononeuritis multiplex; CSF: cerebrospinal fluid; GBS: Guillain-Barré syndrome; MRI: magnetic resonance imaging; RF: rheumatoid factor; ANA Antinuclear antibodies; ANCA: antineutrophil cytoplasmic antibodies; anti-dsDNA: anti-double-stranded DNA; anti-SSA: anti-Sjögren’s-syndrome-related antigen A; anti-SSB: anti-Sjögren’s-syndrome-related antigen B; HIV: human immunodeficiency virus; HCV: hepatitis C virus; CMV: cytomegalovirus; IGRA: interferon gamma release assay; CEA: carcinoembryonic antigen; CA 125: cancer antigen 125; CA 15-3: cancer antigen 15-3; CA 19-9: carbohydrate antigen 19-9.

### Diagnostic Assessment

**Figure 1.**
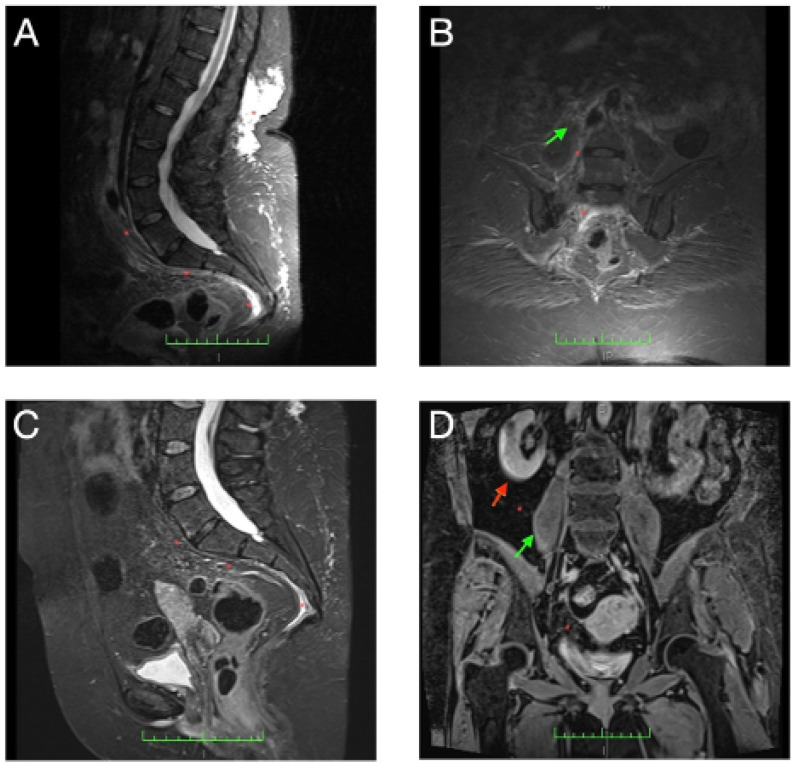
MRI of the lumbosacral spine and pelvis [February (A–B) and April 2019 (C–D)]. **(A)** February 2019-Sagittal T1-weighted fat-suppressed post-contrast MRI of the lumbosacral spine, demonstrating irregular enhancement extending from the lower lumbar levels into the right retroperitoneal and pelvic compartments (red asterisks). Additionally, a focal area of contrast enhancement is visible in the posterior paraspinal musculature at the mid-lumbar level, potentially reflecting spread of inflammation or secondary involvement of adjacent soft tissues. **(B)** February 2019-Coronal T1-weighted fat-suppressed post contrast MRI demonstrating asymmetric enhancement in the right retroperitoneum, with the red asterisks denoting regions of increased post-contrast signal. The green arrow highlights the adjacent right psoas muscle. Subtle surrounding fat stranding is also noted, consistent with an underlying inflammatory or infiltrative process. **(C)** April 2019-Sagittal T2-weighted MRI demonstrating hyperintense signal in the presacral space and anterior to the sacrum, consistent with soft tissue oedema and/or inflammation. Notably, there is substantial resolution of the previously noted retroperitoneal inflammatory changes. **(D)** April 2019-Coronal T1-weighted fat-suppressed post-contrast image reveals asymmetric soft tissue thickening and enhancement in the right retroperitoneal region. Red asterisks denote regions of retroperitoneal fat stranding and subtle soft tissue enhancement. The green arrow points to the right psoas muscle, which shows abnormal enhancement. The red arrow highlights perinephric fat stranding surrounding the right kidney.

Initial laboratory testing, during hospitalisation for abdominal pain from 04–07.02.2019, revealed leucocytosis [white blood cells (WBCs) 15.2×10^3^/μL], with neutrophilia, lymphopenia and eosinopenia. Urinalysis revealed sterile pyuria with haematuria and numerous calcium oxalate crystals. Computed tomography (CT) and magnetic resonance imaging (MRI) (**Figure 1 A–B**) suggested retroperitoneal and pelvic fat stranding with inflammatory infiltration, encasing the right iliac arteries and extending to the aortic bifurcation, with associated fluid adjacent near the uterus and right hip joint. No abscess, ascites, lymphadenopathy, or solid organ involvement were detected. Notably, CT scan revealed no findings of urolithiasis.

These findings suggested an infectious-inflammatory process involving the urinary tract and retroperitoneum. Despite empirical antibiotic therapy, inflammatory markers persisted, with leucocytosis, eosinophilia, and mildly elevated high- sensitivity C-reactive protein (hsCRP 2.89 mg/dl; reference range 0–0.3 mg/dL). Outpatient laboratory work-up (11.02.2019) revealed increased leucocytosis (19.3×10^3^/μL) and erythrocyte sedimentation rate (ESR: 70 mm/h). Urinalysis revealed significant proteinuria, haematuria, mild pyuria and trichomonads but no bacterial growth. Subsequent urine culture grew Escherichia coli and an antibiogram prompted adjustment of antibiotic therapy, though without significant improvement.

During hospitalisation in the neurosurgery department (13–14.02.2019), abdominal CT demonstrated persistent retroperitoneal inflammation, right piriformis muscle swelling, fat stranding extending rightward along the ureter, possible distal right ureterolithiasis, and bilateral renal cortical hypodense lesions suggestive of pyelonephritis, although the patient remained afebrile. Thoracic MRI revealed a large T8–T9 disc herniation with cord compression. Given the initial localised ride-sided deficits and retroperitoneal involvement, primary suspicion revolved around a potential infectious plexopathy, presumptively caused by a lithiasis-related right ureteral injury, extending to adjacent tissues and the lumbosacral plexus. Diabetic neuropathy was also considered but excluded, due to absence of diabetes mellitus (DM) history, normoglycemia and normal hemoglobulin A1c. Subsequent progression involving the left lower limb raised suspicion of Guillain-Barré syndrome (GBS), but cerebrospinal fluid (CSF) analysis and NCS excluded this possibility. CSF analysis (13.02.2019) showed normal cell count, glucose, protein levels and cultures. Oligoclonal bands were not assessed, as there was no clinical sign of central nervous system involvement. This impression was later supported by a normal head CT scan (14.02.2019).

After transfer to the neurology department (14.02.2019), NCS indicated a multifocal asymmetric, motor-predominant axonal neuropathy, affecting the left common peroneal nerve and right sural sensory fibres, a pattern compatible with mononeuritis multiplex. Despite some clinical improvement inflammatory markers peaked (WBC 20×10^3^/μL, ESR 110 mm/h), prompting general surgery and internal medicine consultation, and antibiotic therapy escalation. Moreover, due to persistent retroperitoneum fat stranding and the concern of ongoing inflammation and abscess, gynaecological and urological evaluation were also performed, both without clinically significant findings. A kidney ultrasound and a chest X-Ray showed no abnormal findings. From 22–25.02.2019, WBC declined to 15.8×10^3^/μL, and abdominal CT demonstrated near-complete resolution of fat stranding and pericolic fluid. However, subsequent WBC and ESR increase (18.2×10^3^/μL and 120 mm/h, respectively), prompted recommendation for internal medicine transfer. The patient, however, opted for discharge on 26.02.2019, without a definitive diagnosis. Subsequent abdominal CT scan on 12.03.2019 also demonstrated significant improvement of the previously observed fat stranding.

Follow-up MRI (10.04.2019), demonstrated denervation changes in the right pelvic musculature along the right lumbosacral plexus (L4–S1), with resolution of prior retroperitoneal inflammation (**Figure 1 B–C**). Laboratory tests remained abnormal, with leucocytosis (12–14×10^3^/μL), elevated CRP (27–40 mg/dL; reference range 0–5 mg/dL) and ESR (80–102 mm/h). Serum protein electrophoresis showed hypoalbuminemia with elevated alpha- and beta- globulins, suggesting ongoing systemic inflammation. No monoclonal bands were detected. However, no dedicated cryoglobulin study was performed. Autoantibody and immunologic studies provided unrevealing results, with only a marginally elevated serum complement C4 (57.1 mg/ dL; reference range up to 57.0 mg/dL) being detected, which alone lacks pathologic specificity (**Table 1**). Anti-cyclic citrullinated peptide (anti-CCP) antibodies were not tested. IgG4-related disease (IgG4-RD) was considered in the differential, but deemed unlikely, as total IgG levels were within normal limits (despite IgG4 not being specifically measured), no monoclonal or restricted serum bands were detected on serum protein electrophoresis, and there was no characteristic IgG4-RD organ involvement. However, the absence of histopathologic confirmation – due to the lack of biopsy from the retroperitoneal soft tissues – precludes a definitive exclusion or diagnosis of IgG4-RD. ^9^

The patient was readmitted on 03.05.2019 and a repeated NCS confirmed mononeuritis multiplex, now involving the left median and ulnar nerves and the right sciatic nerve and raised suspicion of an underlying vasculitic aetiology. A right sural nerve biopsy was inconclusive, as the specimen contained insufficient nerve tissue and consisted primarily of surrounding muscle fibres. Additional biopsy, however, was not pursued due to the patient’s wishes. The patient was eventually discharged on 10.05.2019 again without a definitive diagnosis.

### Therapeutic Intervention

The patient received stepwise therapeutic addressing both presumed infection and immune-mediated inflammation. Initially, broad-spectrum empirical antibiotics [intravenous (IV) cefuroxime; oral doxycycline and metronidazole] were started. Treatment was adjusted to oral ciprofloxacin according to the antibiogram, after Escherichia coli isolation in urine cultures on 12.02.2019.

Given the progressive asymmetric deficits up to 13–14.02.2019, immune-mediated neuropathy was suspected. The patient was put on pulse methylprednisolone 1,000 mg/day for 3 consecutive days, and thereafter, due to partial response, she was administered intravenous immunoglobulin (IVIG) at a dose of 2g/kg for 2 days. However, in view of persistently elevated inflammatory markers and concern for occult retroperitoneal infection, immunotherapy was withheld after 48 hours, and antimicrobial therapy was escalated to meropenem and doxycycline.

From 03.05.2019, after MNM diagnosis, long-term oral methylprednisolone (48 mg/day) was initiated, gradually tapered over 12 months, with monthly dose adjustments guided by symptom resolution and serial inflammatory markers (CRP, ESR). The dose was reduced to 10mg/day within 6 months and successfully discontinued at the end of the course. To manage corticosteroid-induced hyperglycaemia, metformin (2 g/day) was co-administered. No additional immunosuppressive agents were used. Concurrently, ciprofloxacin (1 g/day for 7 months) was maintained as chronic prophylactic therapy to mitigate the risk of unresolved low-grade retroperitoneal infection in the context of immunosuppression and persistent inflammatory markers.

### Follow-up and Outcomes

Follow-up CT imaging on 15.05.2019 revealed degenerative changes of the thoracic spine and mild abnormalities in the lower lumbar spine and sacroiliac joint, where sclerosis of the articular surfaces was assessed. Additional serial outpatient laboratory testing demonstrated gradual decline in inflammatory markers, though without complete normalisation. Occasional mild leucocytosis increased inflammatory markers, and mild elevations of liver enzymes and serum urea were attributed to corticosteroid therapy.

At the end of the 12-month regimen, the patient was ambulatory and functionally independent, though with residual moderate motor deficits of the lower extremities and persistent median-nerve-distribution paraesthesias. Six years after 10.05.2019 discharge, she reported slow yet steady improvement, though clinical evaluation did not demonstrate measurable changes compared to the last follow-up after 12 months of therapy.

## LITERATURE REVIEW METHODOLOGY

In accordance with the case-based review standards (CABARET) recommendations, we conducted a comprehensive literature search in PubMed, Scopus, and directory of open access journals (DOAJ) using terms for mononeuritis multiplex (MNM; including synonyms) combined with retroperitoneal pathology (fibrosis, inflammation, disease). This initial search was conducted with the scope of identifying articles where retroperitoneal inflammation was implicated in the pathogenesis of MNM or posed a diagnostic challenge by obscuring the underlying aetiology. No articles fulfilled this criterion. Therefore, we broadened our review to include representative case reports of MNM across aetiologies considered in the differential diagnosis, summarised in **Table 3**. The detailed methodology is summarised in **Supplementary Table 1**. Three additional reports not explicitly documenting MNM were included: two from the Mediterranean Journal of Rheumatology (Maikap et al., Skouvlakidou et al.) to highlight the absence of MNM-focused reports within the journal, and one describing lumbar plexopathy from iliopsoas abscess (Priya et al.) to illustrate how retroperitoneal involvement can focus diagnostic assessment on infectious causes.^10–12^

**Table 3.** Key characteristics of relevant studies.

**Author (Year)**	**Aetiology**	**Patient & Key Clinical Features**	**Diagnostic challenges**	**Treatment**	**Relevance to Current Case**
**Palma et al. (2018)**	HIV with CMV reactivation	MNM during CMV reactivation	Opportunistic infection vs autoimmune neuropathy	ART	Expands infectious spectrum of MNM; illustrates viral trigger
**Macedo et al. (2024)**	EGPA	EGPA with MNM, abdominal pain and systemic symptoms	Absence of characteristic pulmonary and renal manifestations; biopsy confirmation	CS + MTX	MNM as heralding sign in unconventional disease presentation (EGPA)
**Fujii et al. (2012)**	EGPA	Chronic periaortitis within an otherwise typical EGPA presentation with MNM	Periaortitis was attributed to EGPA, though IgG4-RD was also raised as a possible unconfirmed contributor; no nerve biopsy was obtained	CS	Presents a rare retroperitoneal involvement, considered EGPA-related, but with IgG4-RD not definitively excluded, co-existing with MNM
**Chae et al. (2021)**	Systemic vasculitis (non-specific)	Vasculitic MNM complicated by rectal perforation and APS with PE	Not definitive histopathologic confirmation	CS + CYC	Mirrors a complicated case with diagnostic uncertainty and therapeutic management without conclusive biopsy
**Abdelhakim et al. (2022)**	Immune checkpoint inhibitor toxicity	Severe MNM after immunotherapy	Rare iatrogenic cause where broad differential required histology for clarification	IVIg + CS + Rituximab	Adds iatrogenic/oncologic aetiologies; shows drug induced MNM; highlights the role of biopsy in diagnosis by exclusion
**Shi et al. (2023)**	ENKTL	MNM initially misdiagnosed as systemic vasculitis	Initial steroid-responsiveness delayed further investigation; PET findings guided subcutaneous fat biopsy, which revealed lymphoma	DXM + MTX + CYC + Etoposide	Demonstrates neoplastic MNM misdiagnosed as vasculitic neuropathy
**Tanemoto et al. (2020)**	RA with RV	Severe RV-mediated MNM despite long RA remission	RA relapse presenting with vasculitic neuropathy	CS + AZP (+Clopidogrel and Dabigatran for TIA prevention)	Shows MNM may arise in RA even in seemingly quiescent disease
**Bong et al. (2021)**	IgG4-RD	Peripheral neuropathy with MNM features	Renal biopsy confirmed IgG4-RD; mimicked vasculitis	CS	IgG4-RD as important differential in fibro-inflammatory settings
**Maikap et al. (2022) – MJR**	Autoimmune CTD (case series; 1/3 with MCTD and vasculitic neuropathy)	Neuropathic deficits, trigeminal involvement	Rare neurological onset of CTD	CS + CYC	Highlights CTD-related vasculitic neuropathy
**Skouvaklidou et al. (2023) – MJR**	EGPA (case series)	EGPA cases, one multisystemic (respiratory, genital, renal, gastrointestinal) with neuropathy	Known ANCA-negative EGPA with limited discussion of neuropathic spectrum	CS + AZP + Esomeprazole + Spironolactone+ CYC + BDP/FOR + Benralizumab	Neuropathy in multi-systemic EGPA;
**Priya et al. (2024) – Plexopathy**	Iliopsoas abscess (infectious mimic)	Right side LMN deficits and lower back pain, retroperitoneal involvement	Presented as inflammatory neuropathy	Abscess drainage + antibiotics	Parallels retroperitoneal onset in the current patient; highlights infectious mimics

HIV: human immunodeficiency virus; CMV: cytomegalovirus; MNM: mononeuritis multiplex; ART: antiretroviral therapy; EGPA: eosinophilic granulomatosis with polyangiitis; CS: corticosteroids; MTX: methotrexate; IgG4-RD: IgG4-related disease; APS: antiphospholipid syndrome; PE: pulmonary embolism; CYC: cyclophosphamide; IVIg: intravenous immunoglobulin therapy; ENKTL: extranodal natural killer (NK)/T-cell lymphoma; PET: positron emission tomography; DXM: dexamethasone; RA: rheumatoid arthritis; RV: rheumatoid vasculitis; AZP: azathioprine; TIA: transient ischemic attack; CTD: connective tissue diseases; MCTD: mixed connective tissue diseases; BDP/FOR: beclomethasone/formoterol; LMN: lower motor neuron.

## DISCUSSION

To our knowledge, no prior report in the Mediterranean Journal of Rheumatology has focused primarily on mononeuritis multiplex (MNM). Only two publications have noted neuropathic involvement in a relative context – Maikap et al. in a case series of connective tissue diseases and Skouvlakidou et al. in eosinophilic granulomatosis with polyangiitis (EGPA) patients treated with anti-interleukin 5 monoclonal antibodies – but in neither was MNM the central manifestation.^10,11^ The present case instead describes MNM as the leading clinical problem, situated in the context of retroperitoneal inflammation, thereby extending the journal’s literature on neuropathic complications of systemic disease.

Reports of infectious and retroperitoneal processes suggest that they may complicate early evaluation of neuropathic deficits. Priya et al. described an iliopsoas abscess affecting the lumbar plexus, which – though not MNM – paralleled the current patient’s initial unilateral presentation and illustrates how retroperitoneal pathology can direct clinical focus towards an infectious plexopathy.^12^ Palma et al. reported CMV reactivation in an HIV patient presenting with MNM, where distinguishing infection-driven from immune-mediated neuropathy was key in guiding management.^13^ In the current case, early focal deficits, persistent retroperitoneal inflammation and elevated inflammatory markers, led to sustained suspicion of infection, delayed corticosteroids and contributed to prolonged ciprofloxacin prophylaxis being combined with immunosuppression. In vasculitic neuropathies, atypical or nonspecific presentations – like the current case – are not unusual. Macedo et al. described an atypical course of EGPA presenting with MNM, ultimately confirmed through biopsy.^3^ Fujii et al. on the other hand provided a more classic EGPA case, complicated by chronic periaortitis and MNM. While not directly analogous to the current patient – since diagnostic ambiguity was absent – it highlights an unusual occurrence of retroperitoneal involvement and MNM, in the context of systemic vasculitis.^4^ Chae et al. reported MNM caused by an undefined systemic vasculitis, where biopsy – despite not being conclusive – revealed damage patterns strongly suggesting vasculitic neuropathy, and eventually guiding disease management.^5^ Beyond vasculitis, histopathology has also proven pivotal in other contexts: Abdelhakim et al. reported severe MNM secondary to immune checkpoint inhibitor therapy, where biopsy contributed by excluding alternative causes.^6^ Shi et al. similarly described extranodal natural killer/T-cell lymphoma presenting with MNM, initially misdiagnosed as systemic vasculitis and eventually confirmed with abdominal subcutaneous fat biopsy guided by positron emission tomography (PET) findings.^14^ The aforementioned cases underscore how histopathology – even when not definitive or from extra-neural tissue samples – can provide important findings that either confirm or at least narrow the differential. Furthermore, Shi et al. highlight PET’s ability to uncover occult malignancy, a tool that in the present case might have improved diagnostic yield by revealing potential subclinical vasculitis, systemic inflammation or hidden neoplasia.

Beyond vasculitis and malignancy, MNM may complicate autoimmune rheumatic and fibro-inflammatory diseases. Tanemoto et al. reported MNM as a complication of rheumatoid arthritis (RA) despite decades of remission.^15^ This case included serological testing with positive anti-citrullinated protein antibodies (ACPA) and negative cryoglobulins, while in the current case the serological testing didn’t include ACPA. Although this omission remains a limitation, persistently negative serology and long-term clinical stability make it unlikely that ACPA testing would have meaningfully altered the differential. Likewise, the absence of a dedicated cryoglobulin study remains a diagnostic limitation, but the lack of typical systemic features and improvement without antiviral therapy limits the possibility of MNM being associated with cryoglobulinemia.^16^ Bong et al. expanded the autoimmune spectrum by reporting IgG4-RD presenting with MNM, confirmed through renal biopsy and serological testing of IgG4.^7^ In our case however, failure to perform IgG4 levels testing and retroperitoneal tissue biopsy, left the IgG4-RD an unresolved possibility.

The eventual combined corticosteroid-antibiotic therapy reflected the primary diagnostic dilemma between infection and autoimmunity. Although the patient’s response was satisfactory, a confirmed timely diagnosis might have enabled earlier corticosteroid initiation and the addition of agents such as cyclophosphamide, thus potentially improving long-term outcomes and reducing residual deficits.

## CONCLUSION

Our literature search revealed no directly comparable reports of mononeuritis multiplex (MNM), though this is likely due to diagnostic complexity rather than true rarity. Our case illustrates an unusual presentation of MNM with retroperitoneal inflammation, where staggered symptom evolution and overlapping infectious and autoimmune features sustained broad differentials. Investigative gaps contributed greatly to the diagnosis remaining elusive. This highlights the importance of complete autoimmune panels, histopathology (nerve or targeted tissue), and positron emission tomography (PET) imaging in narrowing uncertainty and guiding timely, effective management.
